# The small stellated dodecahedron code and friends

**DOI:** 10.1098/rsta.2017.0323

**Published:** 2018-05-28

**Authors:** J. Conrad, C. Chamberland, N. P. Breuckmann, B. M. Terhal

**Affiliations:** 1 JARA Institute for Quantum Information, RWTH Aachen University, Aachen 52056, Germany; 2 Institute for Quantum Computing and Department of Physics and Astronomy, University of Waterloo, Waterloo, Ontario, Canada N2L 3G1; 3 Department of Physics and Astronomy, University College London, London WC1E 6BT, UK; 4 QuTech, Delft University of Technology, PO Box 5046, 2600 GA Delft, The Netherlands; 5 Institute for Theoretical Nanoelectronics, Forschungszentrum Juelich, 52425 Juelich, Germany

**Keywords:** quantum error correction, fault tolerance, homological quantum codes

## Abstract

We explore a distance-3 homological CSS quantum code, namely the small stellated dodecahedron code, for dense storage of quantum information and we compare its performance with the distance-3 surface code. The data and ancilla qubits of the small stellated dodecahedron code can be located on the edges respectively vertices of a small stellated dodecahedron, making this code suitable for three-dimensional connectivity. This code encodes eight logical qubits into 30 physical qubits (plus 22 ancilla qubits for parity check measurements) in contrast with one logical qubit into nine physical qubits (plus eight ancilla qubits) for the surface code. We develop fault-tolerant parity check circuits and a decoder for this code, allowing us to numerically assess the circuit-based pseudo-threshold.

This article is part of a discussion meeting issue ‘Foundations of quantum mechanics and their impact on contemporary society’.

## 1. Introduction

The popular toric or surface codes are members of a family of topological codes called homological CSS codes [1–3] which can be obtained from tessellations of *D*-dimensional manifolds. Curvature and topology of these manifolds determine features of these codes. Although a code does not specify a specific physical layout or physical distance between qubits, its prescription of which parity checks need to be measured dictates what high-precision interactions need to be engineered between the physical qubits and ancilla qubits for measuring parity checks. As such, a code based on a tessellation of a two-dimensional (2D) flat manifold suits a planar 2D connectivity between qubits, while a three-dimensional (3D) representation of a code in terms of a polyhedron could be used as a template of how physical qubits could be placed and connected up in 3D.

In this paper, we continue the exploration of so-called hyperbolic surface codes [4,5] to determine whether such codes, being block codes with high rate, have advantages over the surface code. The work in [4] constructed various classes of hyperbolic surface codes based on regular tessellations and numerically examined noise thresholds of these codes when subjected to depolarizing noise (assuming noiseless parity checks). The work in [5] went one step further by including effective noise in the parity check measurements themselves, focusing uniquely on {4,5}-hyperbolic surface codes. Breuckmann *et al*. [5] also showed how to do read/write operations using Dehn twists if these block codes are used as a quantum memory. In this paper, we focus on one of the smallest and simplest members of the hyperbolic surface code family, namely a code which has a representation as a small stellated dodecahedron. Going beyond the previous work, we examine the performance of the code when all elementary gates and operations, including those in the parity check circuits, are noisy (more details of the circuit-level noise model are given in §5).

The interest in the small stellated dodecahedron code is that it can pack logical qubits very densely while, like the [[9,1,3]] surface code, still allowing for plain fault-tolerant parity check measurements in combination with a look-up table decoder. Even denser packings of logical qubits in block stabilizer codes are certainly feasible: there are non-CSS codes such as [[8,3,3]], [[10,4,3]], [[11,5,3]], [[13,7,3]] and [[14,8,3]] codes listed in [6]. However, one may expect that the construction of fault-tolerant parity check circuits for such codes requires resource-intense methods such as Steane, Shor or Knill error correction (EC), or flag-fault-tolerance methods [7,8]. Chao & Reichardt [7] also proposed fault-tolerant circuits for a non-topological [[15,7,3]] Hamming code, using only 17 physical qubits in total: a disadvantage of this code is that the weight of the parity checks is high, namely 8, and in the tally of 17 qubits all parity checks are done using the same ancilla qubit.

We find that for a depolarizing circuit-level noise model the stellated dodecahedron code pays for its dense storage with a pseudo-threshold which is a factor 19 lower than that of the Surface-17 code. Despite this somewhat negative message, the methods developed in this paper lay the groundwork for further exploration of these families of codes.

We start the paper by recalling the notion of homological CSS codes, illustrating this code construction by a variety of examples in 2D, representable as star polyhedra, as well as a few 3D and four-dimensional (4D) codes. In §3, we zoom in on the small stellated dodecahedron code, while we zoom out again in §4 by formalizing the problem of optimally scheduling the entangling gates of parity check circuits of LPDC codes or more specifically hyperbolic surface codes. We apply these techniques in §5 to the dodecahedron code obtaining fault-tolerant circuits and describing the decoding method. In §6, we report on the results of our numerical implementation, which includes a direct comparison with the Surface-17 code. We end the paper with a discussion.

## 2. Homological CSS codes

Here, we briefly review the definition of homological CSS codes. We start with a regular tessellation of a *D*-dimensional closed manifold. This defines a *cell complex* composed of *i-cells*, with *i*=0,1,…,*D* referring to the cell dimension. The *i*-cells span a vector space 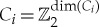 whose elements will be called *i-chains*. Given such a cell complex, one can define a CSS code by associating the *i*-cells with physical qubits, the (*i*+1)-cells with *Z*-checks (i.e. generators of elements in the stabilizer group which only involve Pauli *Z* operators) and the (*i*−1)-cells with *X*-checks (i.e. generators of elements in the stabilizer group which only involve Pauli *X* operators). The number of physical qubits of the code is 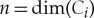. A *Z*-parity check is associated with each (*i*+1)-cell and it takes the parity of the qubits/*i*-cells which form the boundary of the (*i*+1)-cell. Formally, the boundary operator ∂_*i*+1_ is defined as 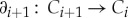. Similarly, a *X*-parity check is associated with each (*i*−1)-cell so that it takes the *X*-parity of all qubits/*i*-cells which are the co-boundary of the (*i*−1)-cell (that is, which have the (*i*−1)-cell as their boundary). Formally, the co-boundary operator *δ*_*i*−1_ is defined as 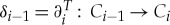. The *X*- and *Z*-parity checks commute because the boundary of any (*i*+1)-cell and the co-boundary of any (*i*−1)-cell overlap on an even number of *i*-cells/qubits.

By the parity check weight of a *X*- or *Z*-parity check, we mean the number of qubits on which this parity check acts non-trivially. The logical *Z* operators (denoted as 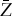) of the code are closed *i*-chains which are not the boundary of any collection of (*i*+1)-cells. Similarly, the logical *X* operators (denoted as 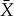) are closed *i*-cochains which are not the co-boundary of any collection of (*i*−1)-cells. The number of logical qubits of the code is given by 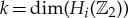 where 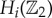 is the *i*th homology group over 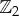, that is 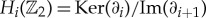. In the next sections, we discuss some concrete code families.

### (a) Two-dimensional hyperbolic surface codes and star polyhedra

Taking a surface (*D*=2), the only choice is for qubits to be associated with 1-cells or edges so that *n*=|*E*|. We only consider regular tilings of the surface. Such tilings can be denoted by the Schläfli symbol {*r*,*s*}, meaning that each face is a regular *r*-gon and *s* of such *r*-gons meet at each vertex. When {*r*,*s*} is such that 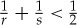, the surface is negatively curved or hyperbolic. For 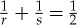 it is flat, and for 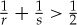 it is positively curved, providing a regular tiling of the sphere. The last choice for {*r*,*s*} gives us all the Platonic solids (e.g. the dodecahedron {5,3}) with trivial topology of the sphere, hence not interesting for encoding quantum information using topology because every closed loop can be contracted to a point.

To make a code out of a hyperbolic surface, one needs to close the surface, so it is topologically equivalent to a many-handled torus. The Euler characteristic *χ* of such a tessellated closed surface equals *χ*=2−2*g*=|*V* |−|*E*|+|*F*| where |*V* |, |*E*| and |*F*| are the number of vertices, edges and faces, respectively, and *g* is the genus of the surface. The surface encodes *k*=2*g* logical qubits. As was argued and reviewed in [1,4], hyperbolic surface codes based on an {*r*,*s*}-tiling have an encoding rate 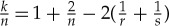 and distance 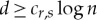 with some constant *c*_*r*,*s*_, which depends on the tessellation.

**Figure 1.**
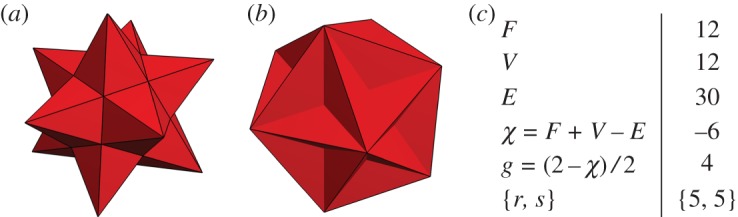
The small stellated dodecahedron as a [[30,8,3]] code (*a*) and its dual polyhedron (*b*) which is called the great dodecahedron. Both polyhedra have the same number of faces, vertices and edges. The vertices of the small stellated dodecahedron lie at the stars where edges meet. With the qubits placed on the edges, the *Z*-checks of the small stellated dodecahedron are described by intersecting pentagrammic faces, denoted as 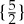. By computing the Euler characteristic *χ* using the parameters in (*c*), it can be seen that the small stellated dodecahedron surface is topologically equivalent to the surface with genus *g*=4. These figures have been typeset using *Stella*. Online available at www.software3d.com/Stella.php.

Some of the smallest codes that one obtains from this construction can be represented as uniform star polyhedra (table 1). Examples are the dodedecadodecahedron based on closing a {5,4}-tiling of the hyperbolic plane [5] with 60 qubits and the small stellated dodecahedron obtained from closing a {5,5}-tiling of the hyperbolic plane, depicted in figure 1. In its representation as star polyhedron, a regular *p*-gon can be represented as a star-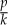-gon (*k* and *p* mutually prime) whose vertices are generated by rotating by an angle 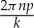 with integer *n* [9]. The Schläfli-notation for a star polygon is 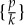, that is, the pentagram is represented as 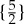 so that the small stellated dodecahedron is denoted as 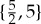.

**Table 1.** Some small uniform star polyhedra with |*E*|=*n* physical qubits, *k*=2*g*= 2−*χ* logical qubits, *Z*- (respectively, *X*) parity check weight wt_*Z*_ (wt_*X*_) and *Z*- (respectively, *X*) distance *d*_*Z*_ and *d*_*X*_. The distances *d*_*Z*_ and *d*_*X*_ were determined using the algorithm described in [5]. A full list of uniform star polyhedra can be found at https://en.wikipedia.org/wiki/List_of_uniform_polyhedra. We omit all uniform polyhedra with *χ*=2, all polyhedra with faces with 10 edges (*Z*-parity check weight 10) and all polyhedra with 120 physical qubits or more. N.O. indicates that the surface represented by the polyhedron is not orientable. As each vertex looks the same (vertex-transitivity) in the polyhedron, all *X*-checks have the same, fairly low, weight and act the same. Except for the small stellated dodecahedron, all dual polyhedra have faces which are not regular polygons (they can be, say, arbitrary quadrilaterals), hence they are not star polyhedra. The many polyhedra with more than one type of polygonal face can also be viewed as quotient spaces of the uniformly tiled hyperbolic plane.

	*n*	*k*=2−*χ*	wt_*Z*_ (wt_*X*_)	*d*_*Z*_ (*d*_*X*_)
tetrahemihexahedron *U*_4_ (a projective plane code)	12	1	3,4 (4)	3 (4)
octahemioctahedron *U*_3_ (a toric code)	24	2	3,6 (4)	4 (5)
cubohemioctahedron *U*_15_ (N.O.)	24	4	4,6 (4)	3 (4)
small stellated dodecahedron *U*_34_ (hyperbolic {5,5})	30	8	5 (5)	3 (3)
great dodecahedron *U*_35_ (dual to *U*_34_)	30	8	5 (5)	3 (3)
small rhombihexahedron *U*_18_	48	8	4,8 (4)	3 (4)
small cubicuboctahedron *U*_13_	48	6	3,4,8 (4)	4 (4)
great cubicuboctahedron *U*_14_	48	6	3,4,8 (4)	4 (4)
great rhombihexahedron *U*_21_ (N.O.)	48	8	4,8 (4)	3 (4)
ditrigonal dodecadodecahedron *U*_41_ (hyperbolic {5,6})	60	18	5 (6)	3 (4) [4]
small ditrigonal icosidodecahedron *U*_30_	60	10	3,5 (6)	4 (4)
great ditrigonal icosidodecahedron *U*_47_	60	10	3,5 (6)	4 (4)
great dodecahemicosahedron *U*_65_	60	10	5,6 (4)	5 (4)
small dodecahemicosahedron *U*_62_ (N.O.)	60	10	5,6 (4)	5 (4)
dodecadodecahedron *U*_36_ (hyperbolic {5,4})	60	8	5 (4)	6 (4) [5]
cubitruncated cuboctahedron *U*_16_	72	6	6,8 (3)	8 (4)

### (b) Some three- and four-dimensional examples based on regular tessellations

If we consider regular tessellations of 3D manifolds, we have the option of placing qubits on edges or faces. As these are dual to each other, one can only construct one code from a given cell complex, so let us imagine that we associate qubits with faces. As 3D manifolds are complicated mathematical objects, it is best to restrict any discussion to concrete 3D cell complexes. A honeycomb is a set of polyhedra filling space such that each face is shared by two polyhedra. We can use the Schläfli-symbol {*p*,*q*,*r*} to denote a regular honeycomb, meaning that *r* regular polyhedra, each of type {*p*,*q*}, meet at a vertex. There is only one regular honeycomb, namely {4,3,4}, a tiling by cubes, which fills flat 3D space and can be wrapped into a 3-torus, hence leading to the 3D toric code.

The 3D versions of the Platonic solids are 6 convex 4-polytopes: examples are {4,3,3} (tesseract) and {5,3,3} (120-cell) and its dual {3,3,5} (600-cell). Instead of filling a flat space, these tile a sphere. In other words, just as the dodecahedron is a regular tiling of the 2-sphere, one can view these cells as regular tilings (by volumes) of the 3-sphere 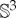. This implies that 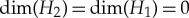, or no qubits are encoded in such objects. The Euler characteristic of these convex polytopes is 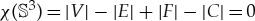 (with |*C*| the number of 3-cells). For example, the 120-cell {5,3,3} has |*V* |=600, |*E*|=1200, |*F*|=720 and |*C*|=120.

Similar to the stellation of a dodecahedron, one can also stellate or greaten a 120-cell or a 600-cell to obtain so-called star polychora with non-trivial topology. An example is the small stellated 120-cell 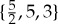 which has |*F*|=720 qubits, |*C*|=120 *Z*-check cells, |*E*|=1200 *X*-check edges and |*V* |=120 vertices, hence its Euler characteristic is *χ*=120−1200+720−120=−480. As 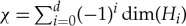 and 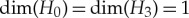, it follows that 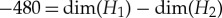, hence allowing for the encoding of logical information.

In 4D, a natural choice is to put qubits on 2-cells, so that one associates a *Z*-check with each 3-cell and an *X*-check with each edge. Beyond the 4D toric code which corresponds to a filling of flat 4D space, namely the honeycomb {4,3,3,4}[10,11], generalizations of the hyperbolic surface codes to 4D are known to exist as well [12,13]. These codes have a number of logical qubits *k* which scales linearly with the number of physical qubits *n*, just like the hyperbolic surface codes. Unlike the 2D hyperbolic codes, the distance of these codes has been shown to scale polynomially with the number of physical qubits *d*∈*O*(*n*^*ϵ*^) with 0<*ϵ*<0.3 [13]. In principle one could create a code starting with a regular tessellation of 4D hyperbolic space by 4-polytopes. To have a closed 4D hyperbolic manifold, one needs to find certain normal, torsion-free subgroups of the Coxeter group [4,14] such that 4-cells related by generators of this group can be identified. One known example is the orientable closed Davis manifold obtained from identifying opposing dodecahedra in the 120-cell, viewed as a 4-polytope [15]. It encodes 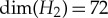 logical qubits and *n*=144 physical qubits (and 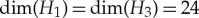) [16].

In [14], an exhaustive search for finding normal torsion-free subgroups of the {5,3,3,3} tessellation of a 4D hyperbolic space is reported. In this tessellation, qubits are associated with pentagons and dodecahedral cells act on 12 qubits. The *X*-checks correspond to tetrahedra in the dual lattice (with Schläfli-symbol {3,3}), having weight 4. Each qubit is acted on by 5 *X*-checks (deg_*X*_=5) and 3 *Z*-checks (deg_*Z*_=3). Unfortunately, running MAGMA to find an exhaustive list of small subgroups of this {5,3,3,3} Coxeter group returns only one quantum code which encodes *k*=197 logical qubits (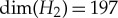) into *n*=16320 physical qubits. For {5,3,3,3} it is the only example which has less than 4×10^4^ physical qubits.

## 3. Features of the small stellated dodecahedron code

**Figure 2.**
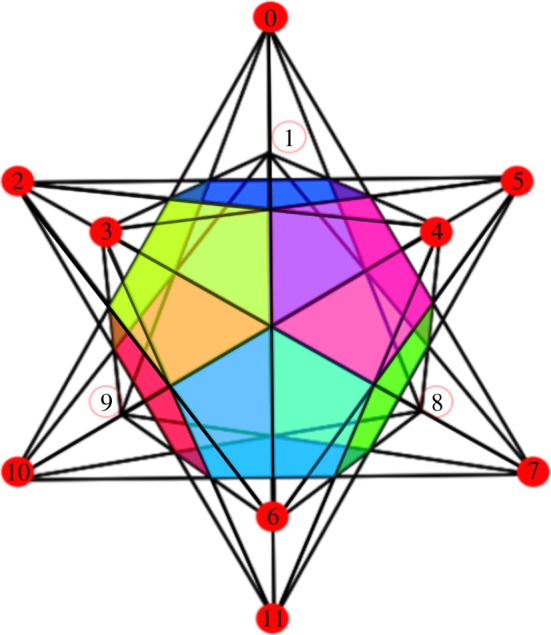
Illustration of the small stellated dodecahedron construction by extending or stellating the edges of the (coloured) regular dodecahedron until they intersect. The labelling of each vertex will be used to identify data qubits as well as the check and logical operators. For example, the check operator localized at vertex 0 is 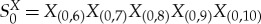.

Some of the features of the small stellated dodecahedron code are summarized in figure 1. The code encodes eight logical qubits (genus 4) into 30 and has distance 3. The *Z*-checks of the code are given by the pentagrammic faces, that is a *Z*-check acts on the five edges of each pentagrammic face. The *X*-checks are located at the vertices, i.e. an *X*-check acts on each of the five edges that meet at a vertex. There are thus 12 *X*- and *Z*-checks each of weight wt(*S*)=5. As the product of all *X*-checks is *I*, the number of independent *X*-checks is 11 (and similarly there are 11 independent *Z*-checks). The small stellated docecahedron is obtained by stellating the dodecahedron as in figure 2, that is, we extend the edges until they meet at new vertices. One can understand the emergence of logical operators due to stellation for this specific polyhedron.^1^

The dodecahedron itself does not encode qubits but this trivial dodecahedron code has qubits on its 30 edges and weight-3 *X* checks and weight-5 *Z*-checks which commute. Now we extend the edges, creating new vertices at which these edges meet. For this new code we keep the weight-5 dodecahedral *Z*-checks and add the weight-5 *X*-checks located at the 12 vertices where the extended edges meet. The 20 weight-3 *X*-checks of the dodecahedron still commute with the *Z*-checks and become possible logical operators.

**Figure 3.**
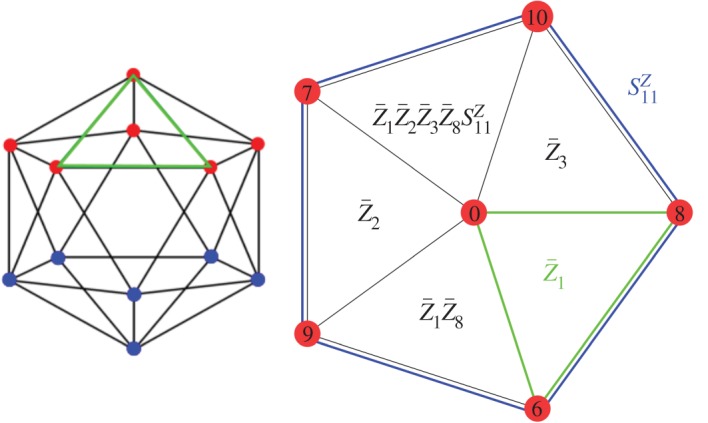
Construction of the logical *Z* operators (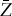) of the small stellated dodecahedron code from its disentangled graph, the icosahedron, where each blue or red vertex corresponds to an *X*-check acting on all incident edges. Each green triangle is a logical *Z* operator commuting with all *X*-checks. In the right figure one sees how a product of 5 logical Z operators, listed in table 2, equals the *Z*-check 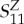. 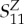 is the *Z*-check associated with the face located above the vertex labelled 11 in figure 2.

In addition, the stellation process creates new weight-3 loops running along a triangle connecting three vertices, and these loops cannot be the product of dodecahedral faces because the edges of the triangle lie in a single plane. When we take the 12 vertices and only represent the edges of the small stellated dodecahedron as a graph, one obtains an icosahedron (figure 3) which allows one to see the linear dependencies between the 20 *Z*-loops. In figure 3, the triangle logical *Z*-operators are represented by the highlighted green edges (any weight-2 *Z* operator would have odd support on at least one *X*-check). Around each vertex the product of 5 of these triangular *Z*-loops is a *Z*-check, hence the number of independent logical *Z*-operators is 20−12=8. Similarly, the 20 vertices of the dodecahedron are logical *X* operators, but products of 5 of them around a dodecahedral face are identical to one weight-5 *X*-check, so there are 20−12=8 linearly independent logical operators. A possible basis for the logical operators is given in table 2.

**Table 2.** Set of independent logical *X* and *Z* operators of the small stellated dodecahedron code obeying 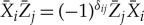. Each qubit is labelled by the edge (*u*,*v*) with vertices *u*,*v* ranging from 0 to 11 as in figure 2.

logical 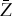 s	logical 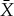 s
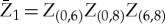	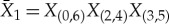
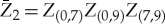	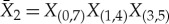
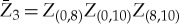	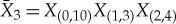
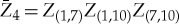	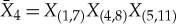
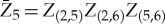	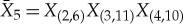
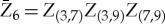	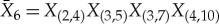
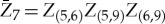	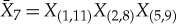
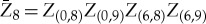	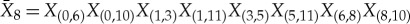

The 3D representation of this code as a small stellated dodecahedron immediately suggests (but does not necessitate) a placement and connectivity of qubits in 3D space. We have also argued in [5] that any hyperbolic surface code can be implemented in a bilayer of qubits with CNOTs required between the two layers while the connectivity between qubits in each layer is planar. Recent experiments on superconducting qubits also demonstrate the feasibility of variable-range planar (hyperbolic) connectivity between qubits [17].

## 4. Parity check scheduling for low-density parity check CSS codes

In general, fault-tolerant EC protocols for LDPC codes are implemented using entangling gates between ancilla and data qubits in order to measure the parity checks. In this section, we assume that parity *X*-checks (respectively, *Z*-checks) are measured via the interaction of a single ancilla qubit with wt(*X*) data qubits via CNOT gates (respectively, wt(*Z*) data qubits via CNOT gates). Thus, at any point in time an ancilla qubit can interact via a CNOT with at most one data qubit. Similarly, any data qubit can interact with at most one ancilla qubit. A relevant problem is to find a scheduling of the CNOT gates which minimizes the number of time steps to measure all parity checks (so as to suppress the occurrence of errors).

This scheduling problem for homological codes based on non-flat geometries is not as trivial as it is for a surface code (or a 4D tesseract code [18]) where a local orientation and order in terms of north, east, south, west can be parallel transported over the whole lattice [19]. This idea does not translate to hyperbolic surface codes since the parallel transport of a vector around a closed curve does not bring it back to itself (in other words, the parallel transported vector depends on the path that one takes) capturing the local curvature. Hence, we formulate the scheduling problem as an optimization problem which can be attacked numerically.

**Figure 4.**
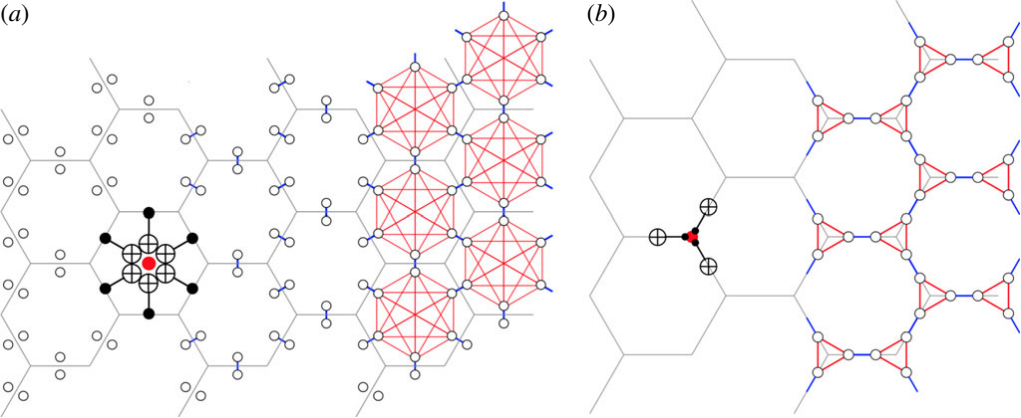
Separate *X* and *Z*-scheduling graphs for the {6,3}-tiling. In (*a*), the scheduling graph for *Z*-checks is shown, whereas in (*b*) the scheduling graph for *X*-checks is shown. In (*a*), each qubit is replaced by two vertices connected by a blue edge. The vertices of all qubits which participate in a hexagonal face are connected by red edges.

For starters, let us imagine that we consider a CSS LDPC (low-density parity check) code and we wish to do all *X*-check measurements with maximal parallelism followed by an optimized schedule for the *Z*-check measurements. In such a scenario, the optimization of the number of time-steps in the measurement of all, say *X*-checks, corresponds to a graph vertex-colouring problem in a graph (to be defined) associated with the LDPC code. This graph and its colouring problem is (non-uniquely) obtained as follows. Each data qubit *q* in the code is replaced by deg_*X*_(*q*) vertices, together forming the vertex set *V*_*X*_ of the *X*-check scheduling graph *G*_*X*_. Here *deg*_*X*_(*q*) is the number of *X*-checks that the qubit participates in, hence the number of CNOT gates that it has to undergo. The edges of *G*_*X*_ are taken as follows. For each qubit *q* we make a clique (complete subgraph) on its deg_*X*_(*q*) vertices, capturing the constraint that none of the CNOT gates are simultaneous. For example, for homological surface codes, we replace each qubit by two connected vertices. Secondly, for each *X*-check of weight wt(*X*) we create a complete graph *K*_wt(*X*)_ between the vertices which represent the qubits on which the parity check acts, capturing the constraint that the CNOTs on the *X*-ancilla qubit cannot act simultaneously. Note that this choice is not unique as each qubit has deg_*X*_(*q*) possible representatives. For homological surface codes, a natural choice which is the same for every edge and face is shown in figure 4. This gives the edge set *E*_*X*_ of the scheduling graph *G*_*X*_=(*V*_*X*_,*E*_*X*_).

Any vertex colouring with *m* colors of the graph *G*_*X*_ gives a schedule which requires *T*=*m*+2 time steps for the *X*-parity check measurements. In other words, the chromatic number *χ*(*G*_*X*_) of the graph *G*_*X*_ determines the number of required time steps. In the first time step, ancilla qubits are prepared in |+〉. In the subsequent *m* time steps, CNOT gates are performed between data and ancilla qubits with the colours of vertices represented by the data qubits labelling the time step at which the CNOT is performed. Note that the colouring assignment only prescribes a temporal ordering up to permutations of time slots. In the last time step, the ancilla qubits are measured. A graph *G* with maximum degree Δ**(*G*) always admits a vertex colouring, i.e. *χ*(*G*)≤Δ**+1 [20]. The degree of *G*_*X*_ is Δ**(*G*_*X*_)=*deg*_*X*_+wt(*X*)−2 when all qubits have the same degree deg_*X*_ and all parity checks have weight wt(*X*). An example for a planar {6,3}-tiling is shown in figure 4. Note that, for {*r*,*s*}-hyperbolic surfaces codes, the degrees of these scheduling graphs are Δ**(*G*_*X*_)=*s* and Δ**(*G*_*Z*_)=*r*.

However, in order to minimize the total number of time steps, it is advantageous to simultaneously apply CNOTs for *X*- and *Z*-check measurements instead of scheduling *X*- and *Z*-check measurements sequentially. Such an *interleaved* schedule has been worked out for the surface code [19,21], leading to a minimal schedule which requires *T*=4+2 time steps (including ancilla preparation and measurement).

**Figure 5.**
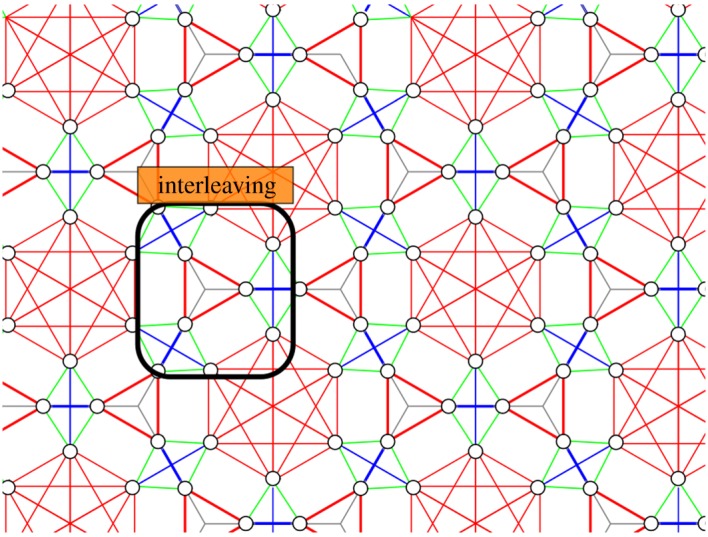
The vertices and edges of the interleaved scheduling graph *G* for a code based on a {6,3}-tiling: one takes the union of the vertices and edges in the graphs *G*_*X*_ and *G*_*Z*_ and adds additional green edges so that each qubit is represented by a clique of four vertices. In the highlighted ‘interleaving’ box, an *X*- and a *Z*-check act on the same pair of data qubits.

Determining an optimal interleaved schedule can again be mapped onto a graph colouring problem obeying an additional constraint which ensures that there is no interference between the two types of measurements. To construct the interleaved scheduling graph *G*, we replace each qubit *q* by deg_*q*_ vertices, where deg_*q*_ is now the total number of parity checks that the qubit participates in. This constitutes the set of vertices *V* . As to the edges, we again make each cluster of deg_*q*_ vertices into a clique. Then we add both the edges of the *X*- as well as the *Z*-checks as we did separately in the graph *G*_*X*_ and *G*_*Z*_. Figure 5 shows the example of the {6,3}-tiling. For codes with qubit degree deg, *X*-parity checks of weight wt(*X*) and *Z*-parity checks of weight wt(*Z*), the degree of this interleaved scheduling graph equals 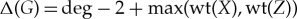. For {*r*,*s*}-surface codes, this results in 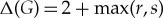 so that 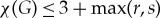 because deg=4. At the same time, the chromatic number 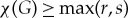 because the graph contains cliques of size *r* and *s*.

However, these colouring-based schedules may not be achievable because the CNOT order is additionally constrained due to the non-commutativity of Pauli *X* and *Z*. To capture this constraint in the colouring problem, one can focus on homological surface codes in which *X*-checks and *Z*-checks have common support on either two or zero qubits (see also another expression of the constraints in [22]).

**Figure 6.**
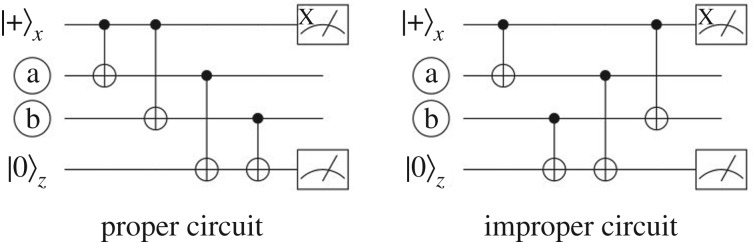
The parity check circuits are proper when, for each pair of qubits a and b which are involved in a *X*- and a *Z*-check, both qubits first interact with the *X*-ancilla and then the *Z*-ancilla or both first with the *Z*-ancilla and then the *X*-ancilla. (*a*) Proper circuit and (*b*) improper circuit.

Consider a pair of qubits, let us call them a and b, on which an *X*- and a *Z*-check both have support (figure 6). Both these qubits have to undergo CNOTs with an *X*-ancilla, as well as CNOTs with a *Z*-ancilla. Irrespective of what other data qubits are involved in the parity check measurements, the outcomes of the two measurements are *proper* when, either both qubits first interact with the *X*-ancilla and then with the *Z*-ancilla or vice versa. In these cases, one can deduce (by propagating Pauli operators through the CNOT gates) that the measurement of *X*_*x*_ of the |+〉_*x*_ ancilla equals the measurement of the observable *X*_*x*_*X*_*a*_*X*_*b*_*I*_*z*_. As the *X*-ancilla is prepared in |+〉_*x*_, this is equivalent to *X*_*a*_*X*_*b*_*I*_*z*_. Similarly, a proper schedule shows that measurement of *Z*_*z*_ is equivalent to measuring *I*_*x*_*Z*_*a*_*Z*_*b*_*Z*_*z*_, which is equivalent to *I*_*x*_*Z*_*a*_*Z*_*b*_ due to the *Z*-ancilla being prepared in |0〉_*z*_. For an improper circuit shown in figure 6*b* the outcome of the parity checks is randomized because the *X*-check measurement depends on the expectation value of *X* on |0〉_*z*_ (and the *Z*-check depends on *Z* on |+〉_*x*_).

There is no general efficient algorithm to find the chromatic number of a graph because the problem is NP-complete. However, for sparse graphs [23] (e.g. Theorem 5) discusses an efficient algorithm under some assumptions. However, our problem is compounded by the additional constraint that the schedule has to be proper. This means that a schedule of 5 rounds of CNOTs for the small stellated dodecahedron code might not be achievable, at least we have not found it. In addition, the schedule is required to be fault tolerant, which puts additional constraints on the schedule. For the small stellated dodecahedron code, we have numerically obtained a sequential non-interleaved *X* and *Z*-parity check schedule which is automatically proper.

We leave the existence of an efficient algorithm for determining a minimal-time parity check schedule for LDPC codes (with sufficiently large distance) as an open question.

## 5. Fault-tolerant circuits for the small stellated dodecahedron code

In this section, we present the fault-tolerant methods that will be used to analyse the performance of the small stellated dodecahedron code. The first step is to find a scheduling of the CNOTs used to measure the checks as discussed in the previous section. By applying a degree of saturation (greedy) algorithm [24], a separate schedule with five colours for both *G*_*X*_ and *G*_*Z*_ could be found (figures 13 and 14). Consequently, all checks can be measured in 10+2 time steps (figure 14). For this schedule we have verified that faults occurring during CNOT gates meet the requirements of fault tolerance (see the discussion in §5a).

We consider the following circuit-level depolarizing noise model for our analysis:

(i) With probability *p*, each two-qubit gate is followed by a two-qubit Pauli error drawn uniformly and independently from {*I*,*X*,*Y*,*Z*}^⊗2^∖{*I*⊗*I*}.
(ii) With probability 2*p*/3, the preparation of the |0〉 state is replaced by |1〉=*X*|0〉. Similarly, with probability 2*p*/3, the preparation of the |+〉 state is replaced by |−〉=*Z*|+〉.
(iii) With probability 2*p*/3, any single qubit measurement has its outcome flipped.
(iv) Lastly, with probability *p*/10, each resting qubit location is followed by a Pauli error drawn uniformly and independently from {*X*,*Y*,*Z*}.

The reason to choose the idling location to have a lower error probability of *p*/10 is that it is a reasonable assumption for actual qubits (such as trapped-ion qubits [25] or nuclear spin qubits around a diamond NV centre [26]) and it brings out more clearly the effect of CNOT errors which dominate the logical failure rate. Taking the idling location to have the same error probability *p* as all other locations would give a disadvantage to the dodecahedron code versus the surface code because the parity check schedule for the dodecahedron code has more qubit idling.

As was shown in [27] (see also the concise description in [8]), a *d*=3 code should obey the following fault-tolerance criteria for an EC unit in order that the logical error probability is possibly below the physical error probability *p*:

**Condition 5.1 Fault-tolerant criteria for an EC unit of a distance-3 codeFault-tolerant criteria for an EC unit of a distance-3 code**

(i) If the input state has *r* errors and the EC unit has *s* faults with *r*+*s*≤1, then ideal decoding of the output state of the EC will result in the same codeword as ideal decoding of the input state.
(ii) Regardless of the number of errors in the input state, if there are *s* faults during the EC unit with *s*≤1, the output state can differ from a valid codeword by an error of at most weight *s*.

Here, ideal decoding means a round of fault-free EC. Furthermore, by a fault we mean any gate, state preparation, measurement or idle qubit failing according to the noise model described above. The second criteria states that if 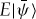 is the input state with codeword 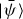 and wt(*E*) is arbitrary, the output state must be written as 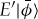 where 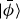 is a codeword and wt(*E*′)≤*s*≤1. Note that it is not required that 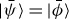.

**Figure 7.**
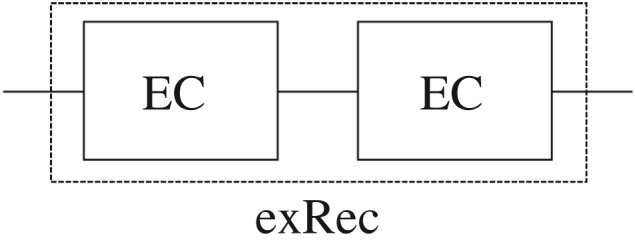
Illustration of an extended rectangle (exRec). The EC unit consists of performing a round of fault-tolerant error correction (in our case, three rounds of syndrome measurements followed by the decoding protocol described in §5a). The exRec consists of performing two consecutive ECs and its logical failure rate is determined by the occurrence of two malignant faults which lead to logical failure on output.

The second condition is particularly important in order to guarantee that errors will not accumulate during multiple rounds of EC resulting in a logical fault. It was shown in [27] (and applied in e.g. [28]) that it is the logical failure probability of an exRec (figure 7), instead of the failure probability of a single EC unit that should be compared to the bare qubit failure probability *p* in order to determine whether the lifetime of an encoded qubit will be longer than that of an unencoded qubit. The reason is that single faults in each consecutive EC unit can lead to logical failure because an incoming error and a fault in the unit can combine together. In the literature, pseudo-thresholds for small distance codes are often computed using a single EC unit. The pseudo-threshold is thus set by the total logical failure probability (probability of a logical *X*, *Y* or *Z* error) of the exRec being equal to *p*. In §6, we explicitly show that the logical failure rate of a single EC cannot be used to estimate the encoded qubit lifetime.

### (a) Decoder for [[30,8,3]]

**Figure 8.**
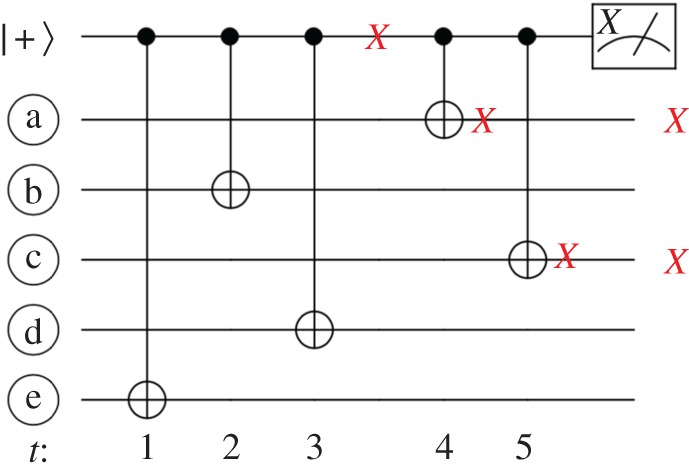
Circuit for measuring a weight-5 *X*-check. A single *X* fault occurring after the third CNOT gate can propagate to two data qubits resulting in two outgoing *X* errors. (Online version in colour.)

As the small stellated dodecahedron code is a distance-3 code, any single data qubit error in the EC unit will be corrected. However, the stabilizer checks are weight 5 which implies, as shown in figure 8, that a single fault occurring on some of the CNOT gates can lead to potentially dangerous errors of weight 2. Note that for any check *P* with wt(*P*)=5, a single fault occurring during its measurement can lead to a data error *E* with weight at most 2 because min(wt_*X*_(*E*),wt_*X*_(*EP*))≤2. Therefore, we need to ensure that any weight-2 errors *E* and *E*′ arising from a single fault during the measurement of an *X* or *Z* check either have a *unique syndrome* compared to each other (*s*(*E*)≠*s*(*E*′)) and compared to single faults which lead to an outgoing weight-1 error, or if *s*(*E*)=*s*(*E*′), then they must be logically equivalent (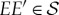 where 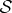 is the stabilizer group).

**Figure 9.**
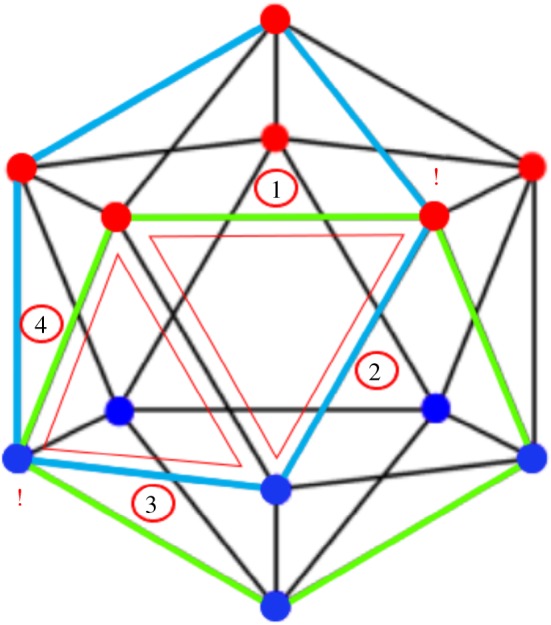
Two pentagonal *Z*-checks, illustrated in green and blue, which overlap on two *Z* logical operators (in red). The pairs of qubits, 1,4 and 2,3 are data qubits which can have *Z* errors arising from a single fault occurring during the measurement of the two checks. The syndromes are indicated by the red exclamation marks.

A useful feature of the code is that the triangular logical *Z* operators in figure 3 overlap with any weight-5 *Z*-check on at most 0 or 1 qubit: a triangular logical *Z* lies in a plane which intersects the pentagrammic planes on at most one edge. However, an example of a problematic scenario involving a product of these logical operators is shown in figure 9. In this scenario, both pairs of qubits 1,4 and 2,3 could undergo *Z* errors arising from a single fault during the measurement of the checks, and note that both pairs of errors have the same error syndrome but *are not* logically equivalent. Thus, correcting the wrong error would lead to a logical fault. To resolve this issue, for the parity check schedule given in figure 13, it was verified that every weight-2 *X*- or *Z*-error arising from a single fault during a stabilizer measurement has a unique syndrome.

With the above considerations, we now describe a decoding protocol which satisfies the fault-tolerant criteria outlined in Condition 5.1. Given the size of the small stellated dodecahedron code, it is possible to decode *X* and *Z* errors separately using full lookup tables (since each contains only 2^11^=2048 syndromes). For a given syndrome *s*, the look-up table chooses the lowest weight error *E* that corresponds to the measured syndrome. However, note that there can be weight-2 errors *E* and *E*′ such that *s*(*E*)=*s*(*E*′) with 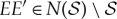 where 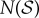 is the normalizer of the stabilizer group. Thus when constructing the look-up table, the corrections associated to all syndromes *s*(*E*) where *E* is a weight-2 error that can arise from a single fault during a stabilizer measurement should be *E* and not some other weight-2 error with the same syndrome as *E* and which is not logically equivalent to *E*. Note that from the above discussion, this look-up table construction is possible.

As with other distance-3 codes, a single round of syndrome measurement is not sufficient to distinguish measurement errors from data qubit errors and would thus not be fault tolerant. To make our decoding scheme fault-tolerant, we use the following protocol:

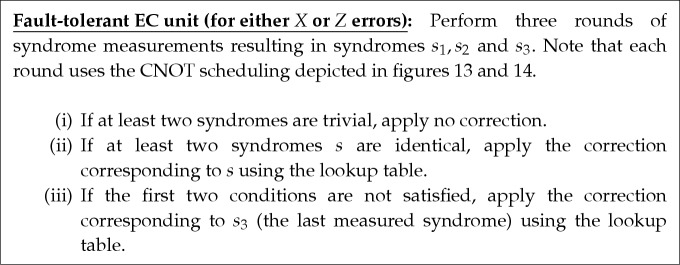


Note that this procedure could be implemented to fault-tolerantly decode any distance-3 code, as long as one can pick a scheduling of the CNOTs (or other entangling gates) that guarantees that all errors arising from a single fault have unique syndromes (and those with the same syndrome are logically equivalent) and one uses these particular errors as the minimum weight corrections in the look-up table.

We now give a rigorous proof that the above procedure satisfies the fault-tolerance criteria of condition 5.1.

First, if there is an input error *E* with wt(*E*)=1 and no faults during the EC rounds, then all three rounds will report the syndrome *s*(*E*) and the error will be corrected. Now suppose there are no input errors but a single fault occurs during the EC. If the fault occurs during the first round, then rounds two and three will produce the same syndrome and the resulting error will be corrected. If the fault occurs during the third round, then the first two rounds will yield a trivial syndrome and no correction will be applied. However, the output error must then be a correctable error. Thus ideally decoding the output would result in the input state. Now if the fault occurs during the second round, then all three syndromes could be different (depending at which time step the error occurred). There is also the possibility that *s*_2_=*s*_3_. In both cases, a correction corresponding to *s*_3_ would be applied removing all errors on the data. Hence, the first criterion will be satisfied.

Lastly, we need to show that the second criterion is satisfied. In fact, we modify the second criterion and demand that the output state differs from a valid codeword by an error which is correctable by our ideal decoder (the ideal decoder is our Look-Up Table Decoder assuming no further errors). As discussed, this could be an error of weight-2. This modification does not alter the use of this condition in deriving fault tolerance [27].

In what follows, we will consider the case where the input error *E* has arbitrary weight. If there are no faults during the EC, then all three syndromes will be equal to *s*(*E*). Hence applying a correction *E*′ based on this syndrome will always project the code back to the code space (i.e. 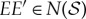). Now suppose there is a single fault during the first round of the EC. Then the syndromes *s*_2_=*s*_3_ will be the syndromes for the combined error *E* and the resulting errors from the single fault during the first round. Thus correcting using *s*_2_ will always project the code back to the code space. If the fault occurs during the second round, then, as in the previous paragraph, the correction will correspond to the last syndrome *s*_3_ which includes both the input error and the error arising from the fault. Thus, correcting using *s*_3_ will always project the code back to the code space. Lastly, if the fault occurs during the third round, then the first two syndromes *s*_1_=*s*_2_ will correspond to the input error *E*. Let *E*′ be the resulting data qubit error from the third round. Then correcting using the recovery 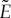 where 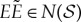, the output state will differ from a valid codeword by an error of weight wt(E’)≤2, which is correctable using our lookup table decoder.

## 6. Numerical results

In this section, we present numerical results for the pseudo-thresholds of the surface-17 code of [21] and the small stellated dodecahedron code using the fault-tolerant decoding schemes and circuit-level noise model presented in §5. To provide a fair comparison, we choose a sequential *X*- and *Z*-check schedule also for the surface-17 code (such a sequential schedule may be a necessity in some architectures anyhow; see e.g. the schedule in [29]). Some of the code can be found at https://github.com/einsteinchris.

To obtain the average lifetime of a physical qubit, suppose that an error is introduced with probability *p* at any given time step. The probability that an error is introduced after exactly *t* time steps is given by *f*_p_(*t*)=(1−*p*)^*t*−1^*p*. Thus the mean time before a failure occurs is given by 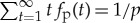. To obtain a lower bound on the lifetime of an encoded qubit, we can simply replace *p* by the logical failure rate curve *p*_L_(*p*) of the exRec (see [27]). For a distance-3 code, *p*_L_(*p*)=*cp*^2^+*O*(*p*^3^) because the code can correct any single qubit error.

**Figure 10.**
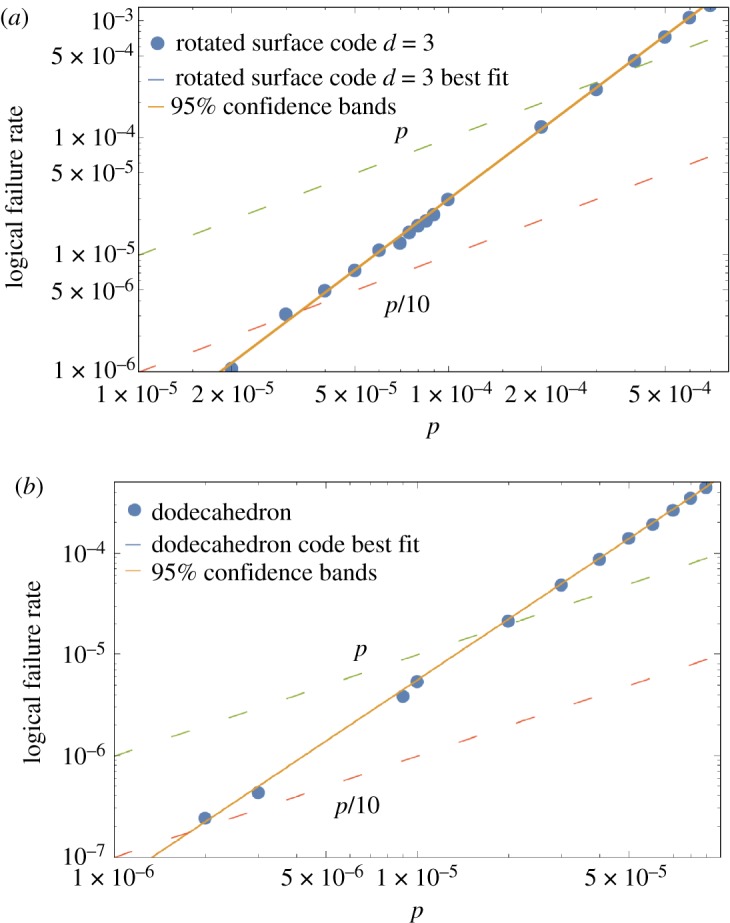
(*a*) and (*b*) show the total logical failure rate (probability of either an *X*, *Y* or *Z* logical fault) curve for the exRec of the Surface-17 (*p*_L_(*p*)≈3000*p*^2^) code and the exRec of the small stellated dodecahedron code (*p*_L_(*p*)≈56488*p*^2^). The intersection between these curves and the curve *f*(*p*)=*p* gives, in principle, the pseudo-threshold of the codes. Note however that since idle qubits fail with probability *p*/10, for quantum memories, the relevant crossover point is given by the intersection with the curve *p*/10 and not *p*. We find that it is (3.32±0.01)×10^−5^ for Surface-17 and (1.77±0.01)×10^−6^ for the small stellated dodecahedron code. The orange lines correspond to the 95% confidence intervals. Since we performed 10^7^ Monte Carlo simulations, the width of the confidence intervals are small and thus overlap with the best fit curve represented by the blue line.

**Figure 11.**
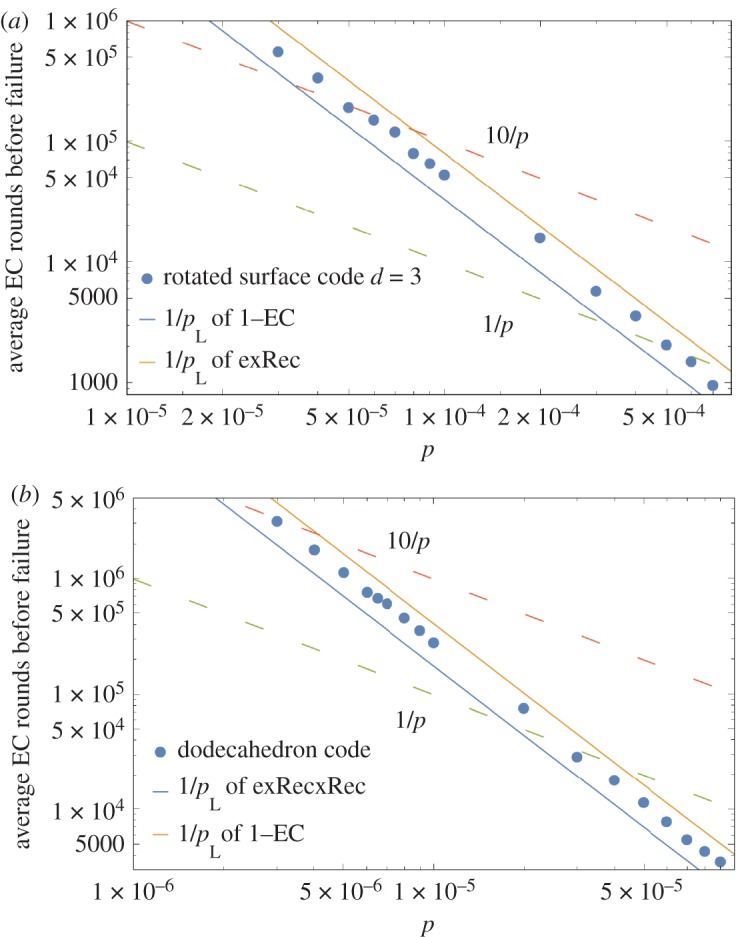
(*a*) shows the average number of EC rounds before failure of an encoded qubit in the surface-17 code, while (*b*) shows the average number of EC rounds before failure of 8 encoded qubits in the small stellated dodecahedron code. Solid lines show 1/*p*_L_ where *p*_L_ is the logical failure rate (as a function of *p*) obtained for both the exRec circuit and the single EC unit circuit. The data clearly show that the lifetime is lower bounded by 1/*p*_L_ obtained from the exRec circuit and not a single EC unit.

In figure 10, plots illustrating the pseudo-threshold of the Surface-17 and the small stellated dodecahedron code are shown. In figure 11, the circular dots show the average number of EC rounds before failure of encoded qubits for both a single qubit encoded in Surface-17 and 8 qubits encoded in the small stellated dodecahedron code (in the simulation, we decoded every three rounds and propagated residual errors into the next EC unit). Unfortunately, the Surface-17 code has a pseudo-threshold which is about 19 times larger than the dodecahedron code ((3.32±0.01)×10^−5^ compared to (1.77±0.01)×10^−6^). Note that the pseudo-threshold values were obtained by the intersection between the curve *p*/10 (because we are considering a noise model where idle qubits fail with probability *p*/10 and are concerned about quantum memories) and the logical failure rate curve of the exRec. The differences in pseudo-thresholds are primarily due to the larger number of locations in the fault-tolerant circuits of the dodecahedron code compared to the surface-17 code circuits as well as the fact that both codes have the same distance. In fact, just by counting the number of pairs of CNOT gates in an EC unit, one can get an indication of the pseudo-threshold. For the small stellated dodecahedron code the number of CNOT gates is 3×5×22=330 so that (330 2) =54285, while for Surface-17 the number of CNOT gates in an EC-unit is 3×4×8=96 so that (96 2) =4560, in rough correspondence with the *c*’s in *p*_L_(*p*)=*cp*^2^ observed in figure 10.

Note that as the dodecahedron code encodes eight logical qubits, a fairer comparison would be to compare the logical failure rate of the dodecahedron code with that of 8 qubits encoded in the surface-17 code. In general, if the logical failure rate of an extended rectangle of the code is given by *p*_L_(*p*), the logical failure rate of *m* copies of the code is given by 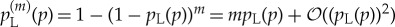.

**Figure 12.**
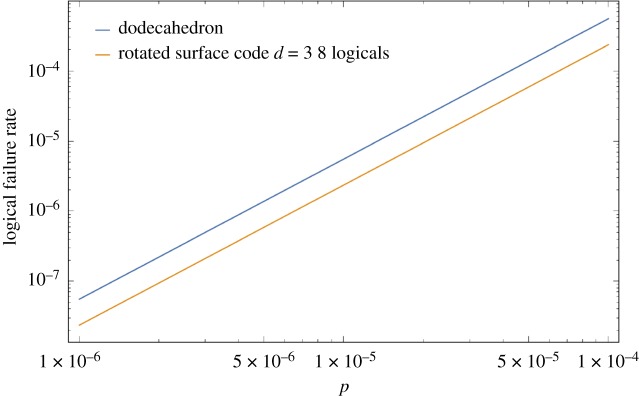
Comparison of eight logical qubits encoded in the surface-17 code, with a total logical failure rate given by 1−(1−*p*_L_(*p*))^8^ where *p*_L_(*p*)≈3000*p*^2^, with eight logical qubits encoded in the dodecahedron code with *p*_L_(*p*)≈56488*p*^2^. It can be seen that the surface-17 still outperforms the dodecahedron code because 8×3000≪56488.

**Figure 13.**
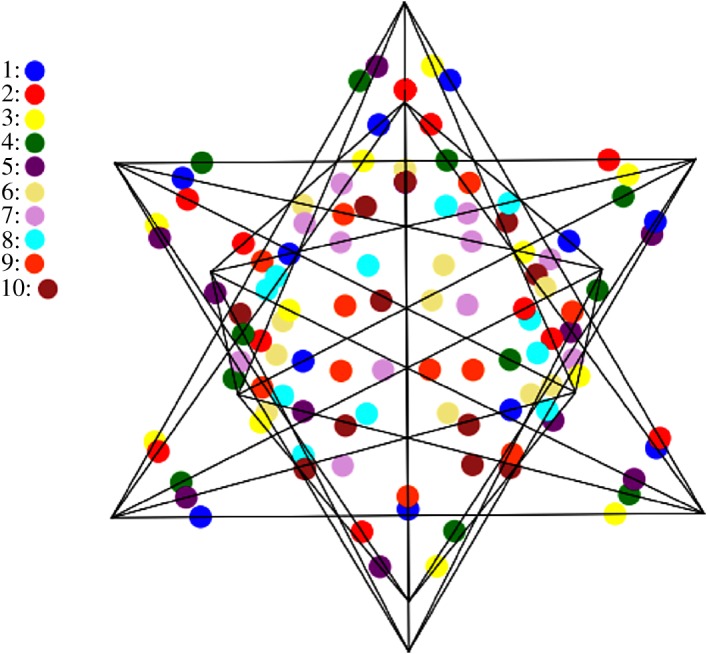
A colouring with 10 colours, see a corresponding schedule in figure 14.

**Figure 14.**
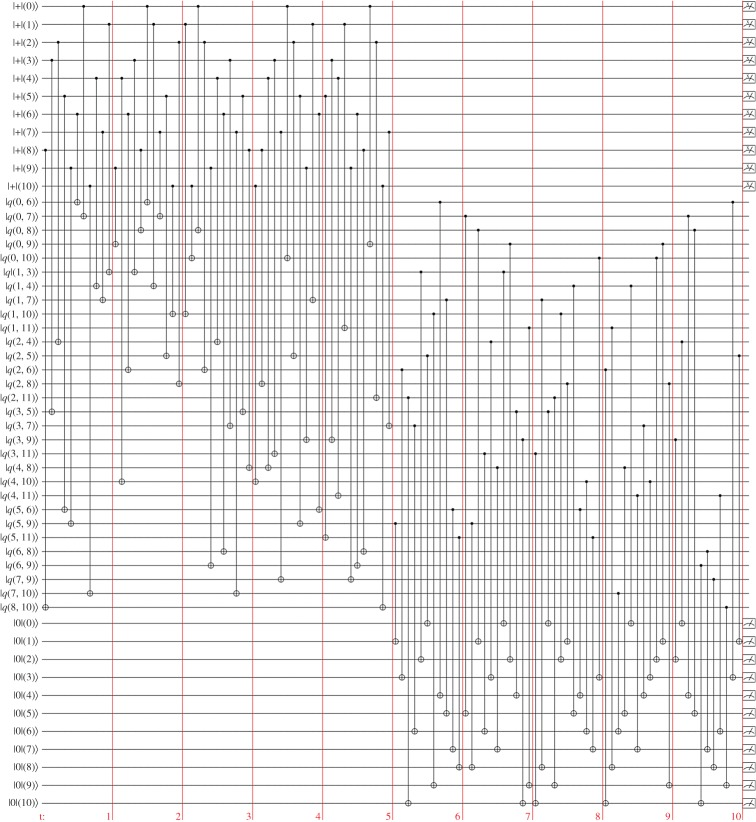
The parity check circuit for one EC round for the small stellated dodecahedron code involving 11 ancilla qubits starting in |+〉 for the *X*-check measurement and 11 ancilla qubits in |0〉 for the *Z*-check measurement. The parity check measurements are accomplished in 10 rounds (separated by red vertical lines), corresponding to a graph colouring with 10 colours.

In figure 12, we compare the logical failure rate of eight qubits encoded in the dodecahedron code with eight qubits encoded in the surface-17 code. It can be seen that the surface-17 code still achieves a lower logical failure rate compared to the dodecahedron code.

## 7. Discussion

The fault-tolerance analysis for the small stellated dodecahedron code has shown the difficulty of getting a block code with high pseudo-threshold when we include noise in the parity check circuits themselves. The EC unit of this code is simply larger because many more checks need to be measured and the pseudo-threshold is determined by pairs of malignant locations in this large unit. By contrast, separate copies of the surface code, each with a much smaller EC unit benefit from having ‘room for each logical operator’. One might expect that this problem becomes less severe for larger hyperbolic codes which have shown lower but still good performance versus surface codes for a phenomenological noise model [5].

One could consider how Steane EC can improve the performance of the small stellated dodecahedron code: we expect that the qubit overhead will be larger (mainly due to the requirement for preparing four logical |0〉 and four logical |+〉 ancillas) but the pseudo-threshold would be quite better. The tetrahemihexahedron code [[12,1,3]] (table 1) with some weight-3 checks might be an interesting variation on the 3×4 rotated surface code (with *d*_*Z*_=3, *d*_*X*_=4).

Lastly, we also tried to use only four of the eight logical qubits of the small stellated dodecahedron code for encoding logical information in order to see if significant improvements in the pseudo-threshold could be obtained. However, our numerical simulations showed that, for various choices of the logical qubits, the pseudo-threshold improved by less than a factor of two. The primary reason is that, in most cases where a failure occurred, several logical qubits were afflicted.

A goal for future work would be to compare the performance of the small stellated dodecahedron code with the surface code for a physically motivated noise model in an optically linked ion-trap architecture [30] or an optically linked NV-centre in diamond architecture [31].

## Footnotes

1 For polyhedra one can also extend faces instead of edges; this is called greatening. An example is the greatening of the octahedron into the stella octangula. As qubits are not defined on faces, this process does not create an interesting code. One can stellate the icosahedron into the small triambic icosahedron (which is dual to *U*_30_; see table 1), but there does not seem to be an interpretation of stellation as creating non-trivial topology in this case.

## Data accessibility

This article has no additional data.
